# Supplementary material for: Development of a Training Strategy Aimed at Increasing Veterinarians’ Awareness of the Proper Use of Antibiotics on Rabbit Farms
Source: Animals (Basel). 2023 Jul 26;13(15):2411. doi: 10.3390/ani13152411 (PMC10416837; doi:10.3390/ani13152411)
Supplement: Supplementary file 1 [file animals-13-02411-s001.zip › S1 The operational tool.pdf]

Table S1: The operational tool

1 - Characteristics of breeding and its management

1. Housing and prophylaxis

Biosecurity measures	Indirect prophylaxis	Two cycles in the same housing facility	*notes*
		Regular disinfection	
		Rodent control program	
		Pest control program	
		Presence of nets against birds	
		Cleaning and removal of manure under cages	
		Manure management	
		Carcass management (removal and disposal)	
	Movement of vehicles and people	Presence of vehicle disinfection system (e.g. arch)	
		Presence of a disinfection area	
		Filter zone for changing clothes	
		Outside silos for unloading feed	
		Entry register	

2. Nutrition management

Feed	Feed stocking	Double silo use	*notes*
	Pellet composition	Differentiated grinding of fibrous part	
	Feeding program	Separation of medicated and non-medicated feed	
		Restricted	
		Ad libitum	
	Presence of undesirable substances	Penicillins	
		Ionophores	
		Mycotoxins	
Water	Source	Well bored into underground aquifer (known water chemical and physical characteristics)	
		Aqueduct (known water hardness)	
	Distribution system	Purification systems for well water	
		Water tanks and watering lines sanitation (frequency, incrustation and mucilage removal check)	

3. Breeding sector

	Introduction of new breeders	The absence of pathology is certified		Management of new breeders	Housing system management	Clean housing system	*notes*
	Semen purchase	The absence of pathology is certified				Cage type	
	Critical production moments	Sanitation				Type of ventilation, heating, watering systems	
		Weaning				Stocking density	
		Loading			Introduction of new animals	Quarantine cages	
		Before kindling				Prophylaxis	
	Physical hygiene methods with animals in the farm	Removal of manure			Feeding	Adequate daily feed intake	
		Pressure washer with warm water				Controlled feed intake	
		Pressure washer with detergents				Weight at first insemination	
	Physical hygiene methods without animals in farm	Blowers			Indirect prophylaxis	Before contacting other animals, future parents are vaccinated and housed in quarantine rooms	
		Pressure washer with warm water				Registered vaccine administration	
		Pressure washer with detergents				Autogenous vaccine administration	
		burners for cages and surfaces			Productive rhythm	Single band	
Microclimate	Risk factors for disease onset	Quick temperature changes				Dual band	
		Warm and humid		Good insemination practices	Hygiene of equipment	Correct use of single-use material (e.g. needles.)	
	Control of microclimat	Carbon dioxide			Semen purchase	Certified absence of pathology	

	e parameters (air speed, cooling, etc.)	Ammonia			Hormone management	Correct hormone dosage	
		Temperature				Heat induction hormones	
		Humidity				Ovulation induction hormones	
	Control systems	Sensors and control units		Kindling and nest managem ent	Hygiene	Setting up the nest with sterile materials	
						Cleanliness of the nest	
					Litter balancing	By parity order	
						Primiparous management	

2 - Diseases detectable on the farm

Diagnosis and selection of the most appropriate intervention

1. Fattening sector: gastrointestinal pathologies

		notes	Interve-ntion
Parameters considered	Mortality		
	Morbidity		
	Temperature		
	Animals' age		
	Number of days to slaughter		
	Decrease in food consumption		
Sampling	Sample type		
	Sample time (days after the problem onset)		
	Ongoing medication influence on bacteriological investigations		
	Sample size		
	Sampling points within the shed		

2. Fattening sector: respiratory and integumentary system pathologies

		notes	Interve-ntion
Parameters considered	Mortality		
	Morbidity		
	Visit (animal and environment checks)		
	Farm anamnesis		
	Drugs used		
	Number of days to slaughter		
Laboratory analysis	Anatomopathological examination		
	Bacteriological tests		
	Respiratory and/or integumentary panel of tests		
	MIC		
	Any additional test		

3. Breeding sector (reproducing does): respiratory and integumentary system pathologies

		notes	Interve-ntion
Considered parameters	Parity order		
	Reproductive cycle phase		
	Morbidity		
	Visit (animal and environment checks)		
	Farm anamnesis		
	Drugs used		
Laboratory tests	Bacteriological tests		
	Strain characterization		
	MIC		
	Any additional test		
Sampling	Sample type		

	Sample transport to the laboratory				Sample type				Sample size		
	Anatomopathological examination			Sampling	Sample size				Sampling points within the shed		
Laboratory tests	Bacteriological tests				Sampling points within the shed				Sample transport to the laboratory		
	Parasitological tests				Sample transport to the laboratory						
	MIC										
	Any additional test										
