# Supplementary material for: Development of a Training Strategy Aimed at Increasing Veterinarians’ Awareness of the Proper Use of Antibiotics on Rabbit Farms
Source: Animals (Basel). 2023 Jul 26;13(15):2411. doi: 10.3390/ani13152411 (PMC10416837; doi:10.3390/ani13152411)
Supplement: Supplementary file 1 [file animals-13-02411-s001.zip › S2 The practical exercise sheet.pdf]

Table S2: The practical exercise sheet

**Practical exercise**	
1. CHARACTERISTICS OF THE FARM AND ITS MANAGEMENT	
Briefly describe the characteristics of the farm (type, location, number of animals, type of systems, management methods, etc.) you will consider for the exercise.	____________________<br>____________________<br>____________________
2. ISSUES FOUND	
Describe the problem that was identified in the farm; provide as much information as possible (number of animals involved, systems involved, any similar cases previously verified).	____________________<br>____________________<br>____________________
3. COMMENTS AND PROPOSALS FOR INTERVENTION	
Using the operational tool, explain your observations with respect to the critical aspects identified and formulate your intervention proposal by referring to all the variables that, according to your professional experience, play a role in the problem or in its solution.	Structural/management interventions (see Appendix 1)<br>____________________<br>____________________<br>Therapeutic interventions (see Appendix 2)<br>____________________<br>____________________
